# The frequency of bowel and bladder problems in multiple sclerosis and its relation to fatigue: A single centre experience

**DOI:** 10.1371/journal.pone.0222731

**Published:** 2019-09-19

**Authors:** Sophia D. Lin, Jane E. Butler, Claire L. Boswell-Ruys, Phu Hoang, Tom Jarvis, Simon C. Gandevia, Euan J. McCaughey

**Affiliations:** 1 Neuroscience Research Australia, Randwick, NSW, Australia; 2 School of Medical Sciences, University of New South Wales, Kensington, NSW, Australia; 3 Continence Clinic, MS Limited, Sydney, Australia; 4 Australian Catholic University, Sydney, Australia; 5 Prince of Wales Clinical School, University of New South Wales Medicine, University of New South Wales, Sydney, NSW, Australia; University of Melbourne, AUSTRALIA

## Abstract

**Background:**

Bowel and bladder problems affect more than 50% of people with Multiple Sclerosis (MS). These problems have a large impact on quality of life and place a significant burden on health systems.

**Objectives:**

This study aimed to ascertain the frequency of bladder and bowel problems in a select Australian MS cohort and to investigate the relationships between level of disability, bladder and bowel problems, and fatigue.

**Methods:**

Questionnaires on the nature and severity of MS symptoms were distributed to clients attending an Australian MS centre. Log-binomial regression and multiple linear regression models were used to investigate relationships between disability, fatigue, and bladder and bowel problems.

**Results and conclusions:**

Of 167 questionnaires distributed, 136 were completed. Bladder problems were reported by 87 (74.4%) respondents, whilst 66 (48.9%) experienced functional constipation and 43 (31.9%) faecal incontinence. This frequency in our select Australian MS population is similar to that reported globally. There was a significant correlation between level of disability and: bladder problems (p = 0.015), faecal incontinence (p = 0.001), fatigue (p<0.001) and constipation (p = 0.016, relative risk: 1.16). Further investigation into the causal relationships between various MS symptoms may be beneficial in the development of novel therapeutic strategies for people with MS.

## Introduction

Multiple sclerosis (MS) is a life changing disease affecting approximately 2.3 million people globally, with prevalence continuing to increase.[1] In Australia, estimated prevalence of MS is 23,700.[2] The impact of MS is profound, affecting the individual physically, mentally and financially. It is associated with a poorer quality of life and results in considerable costs to both the individual and the community.[1]

The clinical course and presentation of MS varies between individuals, and is dependent on the location of the lesion or plaque.[3] Common symptoms include muscle weakness, coordination problems, impaired sensation and motor function, visual deficits, fatigue, and bladder or bowel dysfunction.[4] Central Nervous System (CNS) lesions that disrupt nervous pathways between the pons and the sacral nerves give rise to urinary symptoms such as increased urgency, frequency, urge incontinence, voiding dysfunction and urinary retention.[5, 6] Demyelinating CNS lesions can result in slow colonic transit, decreased rectal sensation and contractile response. In addition, MS-related factors such as use of anticholinergics or antispasmodics can also lead to chronic constipation and faecal incontinence.[7, 8] The impact of these bowel and bladder problems is significant, and is one of the most common problems that limit the ability of people with MS to work and remain employed.[9] They can also result in social inconvenience and isolation, decreased quality of life and increased morbidity.[10–13] Furthermore, the financial costs associated with MS-related bowel and bladder dysfunction are substantial.[11, 13]

Despite the relatively high prevalence of bowel and bladder problems in MS, there is limited data regarding bowel and bladder dysfunction in the Australian MS population. So far, the only published data is from the states of Victoria and Queensland.[11, 13] Furthermore, while several studies have attempted to document the prevalence of bowel and bladder dysfunction in MS populations in other parts of the world,[7, 12, 14] few have explored the possible relationship between bladder and bowel problems and other concurrent MS symptoms such as disability and fatigue.[1, 13, 15, 16] In particular, the gastrointestinal system and its associated pathology in people with MS has received scant attention as compared to the urinary system, and extensive research on MS-related bowel dysfunction is lacking.[10, 13, 14] The aim of this study is to investigate the frequency of bowel and bladder symptoms in a selection of the MS population in New South Wales, and to explore the relationship between the most frequently reported symptoms in the Australian MS population–fatigue, disability, and bowel and bladder problems.

## Methods

### Study design

This prospective survey based study was conducted at Neuroscience Research Australia, Sydney, Australia. Questionnaires enquiring on bowel, bladder and fatigue symptoms were distributed to clients who visited the Studdy MS centre, Lidcombe, Australia, during an 8-month period from March to October 2018. Studdy MS centre is a general-purpose MS centre that offers a variety of services for people with MS, including symptom management (involving doctors and nurses), a gymnasium staffed by physiotherapists, educational programs and social work services. A convenience sample was made from all clients who visited the Studdy MS centre on 20 days during the study period were approached to participate in the study. Ethics approval was granted by the University of New South Wales Human Research Ethics Committee (Local Code; HC180005) and all participants provided written informed consent.

People with clinically confirmed MS were eligible for inclusion if they were at least 18 years of age, able to provide informed consent, and able to provide either verbal or written responses to the questions in the survey. Basic demographic information including age, gender, year of diagnosis of MS, type of MS, EDSS (Expanded Disability Status Scale) score,[17] and medical co-morbidities were included in the questionnaire and documented. All data were self-reported. In particular, EDSS scores were participant-reported, without comparison to medical records. As such, the contribution of the functional systems (not just ambulation) to the EDSS cannot be discriminated based on this self-report.

### Study tools

#### Actionable bladder symptom screening tool

The simplified, 8-item Actionable Bladder Symptom Screening Tool (ABSST), a validated and accurate measure of the severity of neurogenic bladder problems in people with MS,[18, 19] was used to assess bladder symptom severity such as urgency, urinary leakage, frequency of micturition, nocturia, and the psychosocial impact of bladder problems. It has a maximum score of 24, with a cut-off score of 6 shown to have a high sensitivity and specificity in the detection of bladder problems that are significant enough to warrant urological referral in the MS population.[20]

#### Rome III criteria and Revised Faecal Incontinence Scale (RFIS)

The Rome III diagnostic criterion for functional gastrointestinal disorders was used to assess the frequency of constipation in our population. [21] The Revised Faecal Incontinence Scale (RFIS), a validated measure of faecal incontinence, [22, 23] was used to assess the severity of faecal incontinence and stool leakage. It has a total score ranging from 0 to 20; a score <4 indicates that there is no faecal incontinence whilst a score of 4–6 suggests mild faecal incontinence, scores of 7–12 moderate faecal incontinence, and scores >12 indicate severe faecal incontinence.[24]

#### Modified Fatigue Impact Scale (MFIS)

The Modified Fatigue Impact Scale (MFIS) is a 21-item questionnaire, developed and adapted from the internationally recognised and well-validated 40-item Fatigue Impact Scale (FIS).[25, 26] It has a total score of 84, and was used to assess the severity of fatigue symptoms in the 4 weeks prior to completion of the questionnaire.

### Data analysis

Responses to the questionnaire were initially collected on paper and the data entered in to the REDCap electronic data capture tool.[27] A total of 167 questionnaires were distributed, with 136 (81.4%) returned. Descriptive statistics of participant demographics (n = 136) were generated and the frequency of significant bladder, bowel and fatigue-related symptoms were classified according to EDSS group using IBM SPSS (version 25, IBM, NY, USA). Permanent indwelling catheter use was documented in 18 responders, with the short form ABSST not applicable to this group. From the remaining 118 responses to the ABSST, one participant had an unknown EDSS score and was thus excluded from the analysis.

**Fig 1:**
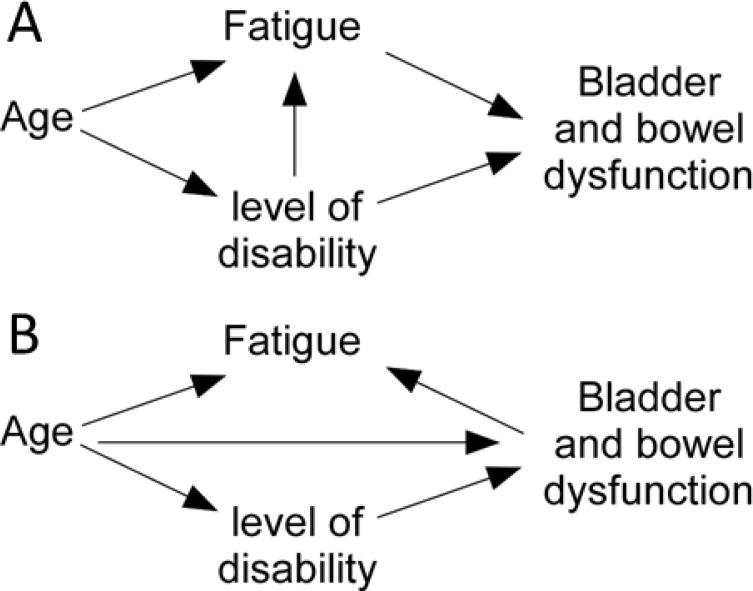
Causal directed acyclic graphs to determine variables that may need adjusting for in regressions designed to investigate the relationship between fatigue, level of disability (as measured by Expanded Disability Status Score), and bowel and bladder problems in people with Multiple Sclerosis. Fig 1A investigates the impact of fatigue and level of disability on bowel and bladder function, while Fig 1B investigates the impact of bowel and bladder dysfunction on fatigue.

Log-binomial regressions were carried out to examine the relationship between EDSS and fatigue (MFIS) scores, and functional constipation. Multiple linear regression models were used to investigate the relationship between EDSS and fatigue (MFIS) scores with bladder dysfunction (ABSST scores) and faecal incontinence (RFIS scores). Causal directed acyclic graphs (DAG) were devised in Dagitty (online version 2.3) and for each model, variables that that were determined to be potential confounders were identified and adjusted for in the multiple linear or multiple logistic regressions. The models used are shown in Fig 1. When looking at the causal relationship between level of disability (EDSS score) and measures of bladder and bowel dysfunction, age was adjusted for by including it in the regression (Fig 1A). When looking at the causal relationship between fatigue and bladder and bowel dysfunction, EDSS was adjusted for by including it in the regression. A multiple linear regression was also performed to investigate the causal relationship between level of disability (EDSS score) and fatigue, by adjusting this relationship for age. Because of the uncertainty of whether fatigue would cause bladder and bowel dysfunction or whether bladder and bowel dysfunction would cause fatigue (see Discussion), a second DAG was used to investigate the reverse relationship (Fig 1B), where the causal relationship between bladder and bowel dysfunction and fatigue was investigated, adjusted for age. All results are reported as mean (± standard deviation) unless otherwise stated and a p-value <0.05 was considered statistically significant. All analysis was performed using IBM SPSS (version 25, IBM, NY, USA). All raw data used for analysis is provided in S1 Table.

## Results

A total of 167 questionnaires were distributed, with 136 completed and returned (81.4% response rate). No data was collected relating to the 31 participants who declined to participate. Demographics of the study population are outlined in Table 1. The majority of responders had EDSS scores between 5–7.5, suggesting a moderate level of disability.

**Table 1:** Demographic information and baseline clinical characteristics of study population (n = 136). ABSST: Actionable Bladder Symptom Screening Tool [19]; EDSS: Expanded Disability Status Scale [17]; MFIS: Modified Fatigue Impact Scale [26]; RFIS: Revised Faecal Incontinence Scale [22]; SD: Standard Deviation.

**Age**	
Mean (SD)	56.1 (12.3)
Range	25–81
**Gender**	
Male	46/136 (33.8%)
Female	90/136 (66.2%)
**Type of MS**	
Relapse Remitting	44/136 (32.4%)
Secondary Progressive	58/136 (42.6%)
Primary Progressive	27/136 (19.9%)
Unknown	7/136 (5.1%)
**No. of years since diagnosis**	
Mean (SD)	18.2 (10.8)
Range	1–43
**Age of MS onset**	
Mean (SD)	38.0 (13.0)
Range	9–69
**EDSS scores**	
0–4.5 (fully ambulatory)	37/136 (27.2%)
5–7.5 (ambulatory with aid)	84/136 (61.8%)
8–10 (non-ambulatory)	14/136 (10.3%)
Unknown	1/136 (0.7%)
**ABSST scores**	
Mean (SD)	8.52 (4.66)
Range	0–21
**RFIS scores**	
Mean (SD)	2.85 (3.97)
Range	0–18
**MFIS scores**	
Mean (SD)	39.7 (19.1)
Range	0–77

### Bladder dysfunction

A cut-off ABSST score of 6 was used to indicate significant bladder dysfunction. 74.4% (87/117) of all responders without an indwelling catheter had an ABSST score ≥ 6.

It is reasonable to assume that all 18 responders that had a permanent indwelling catheter had significant bladder dysfunction, as permanent catheterisation is often employed in the management of bladder problems only after conservative and non-invasive management strategies have failed.[28, 29] Taking this into account, a total of 105/135 responders (77.8%) experienced some form of bladder problems. An additional section enquiring on symptoms related to urinary incontinence (UI) amongst participants who had experienced urinary leakage revealed that urge incontinence was the most common form of UI (71.11%). Other forms of UI experienced included stress incontinence (14.44%) and mixed incontinence (8.89%). The type of UI was unknown in 5.56% amongst those who experienced UI.

**Fig 2:**
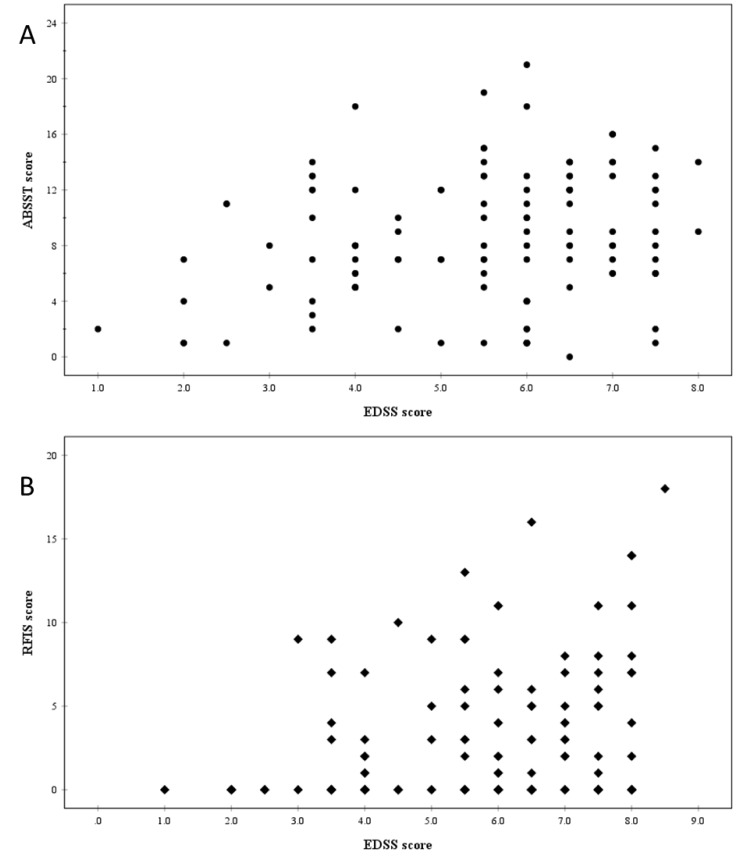
Expanded Disability Status Scale (EDSS) [17] scores of participants with Multiple Sclerosis plotted against A, Actionable Bladder Symptom Screening Tool (ABSST) [19] scores (n = 117) and B, Revised Faecal Incontinence Scale (RFIS) [21] scores (n = 135).

A multiple linear regression model showed a significant relationship between EDSS (level of disability) and ABSST (severity of bladder problems) (p = 0.015), with little effect of age (Table 2). Individual scores are presented in Fig 2A.

**Table 2:** Relationship between level of disability, bladder dysfunction, bowel dysfunction, and fatigue. EDSS: Expanded Disability Status Scale (range 1–10); MFIS: Modified Fatigue Impact Scale (range 1–84); ABSST: Actionable Bladder Symptom Screening Tool (range 1–24); RFIS: Revised Faecal Incontinence Scale (range 1–20).

Effect	Putative cause	Adjusted for	Effect per unit cause	95% CI
Bladder dysfunction (ABSST)*	Disability (EDSS)	Age	0.7	0.1 to 1.2
Bladder dysfunction (ABSST)*	Fatigue (MFIS)	Disability (EDSS)	0.10	0.05 to 0.14
Faecal incontinence (RFIS)*	Disability (EDSS)	Age	0.7	0.3 to 1.1
Faecal incontinence (RFIS)*	Fatigue (MFIS)	Disability (EDSS)	0.06	0.03 to 0.10
Fatigue (MFIS)**	Disability (EDSS)	Age	3.6	1.7 to 5.5
Fatigue (MFIS)**	Bladder dysfunction (ABSST)	Age	1.7	1.0 to 2.3
Fatigue (MFIS)**	Faecal incontinence (RFIS)	Age	1.6	0.9 to 2.4

* Refers to Causal directed acyclic graphs in Fig 1A.

** Refers to Causal directed acyclic graphs in Fig 1B.

### Bowel dysfunction

48.9% (66/135) of respondents fulfilled at least 2 of the criteria listed in the Rome III diagnostic criteria for functional constipation. RFIS scores indicated about 31.9% (43/135) of respondents presented with some form of faecal incontinence.

A log-binomial regression showed a significant positive relationship between EDSS scores and the presence of functional constipation (defined by the Rome III criteria), adjusted for the confounding effects of age (p = 0.018). For a person aged 20 with an EDSS of 1, the baseline probability (risk) of constipation was 24% (0.238 (95% CI, 0.112 to 0.506)). The risk of constipation increased by 16% for every 1 point increase in EDSS (1.164 (95% CI, 1.027 to 1.320)). Note, there was no effect of age (p = 0.987).

A multiple linear regression model also showed a significant positive relationship between level of disability (EDSS) and severity of faecal incontinence (p = 0.001), adjusted for age (Table 2). A scatter plot of individual EDSS and RFIS scores, representing disability and faecal incontinence, is presented in Fig 2B.

### Fatigue

**Fig 3:**
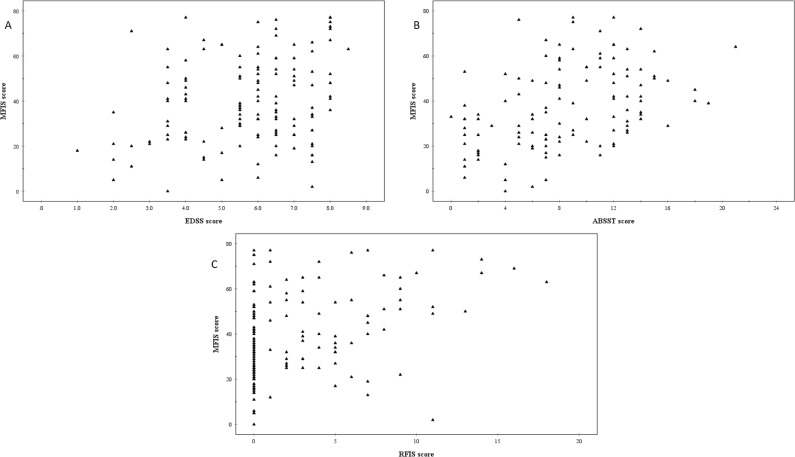
Individual participants’ Modified Fatigue Impact Scale (MFIS) scores plotted against A, Expanded Disability Status Scale (EDSS) scores (n = 135), B, Actionable Bladder Symptom Screening Tool (ABSST) scores (n = 117) and C, Revised Faecal Incontinence Scale (RFIS) scores (n = 135).

The MFIS scores of individual participants were positively correlated with level of disability (p<0.001), adjusted for age (Fig 3A, Table 2).

#### Bladder dysfunction and fatigue

There was a significant positive correlation between fatigue and the severity of bladder dysfunction (p<0.001, Table 2), adjusted for level of disability. Similarly, there was also a significant positive relationship between the severity of bladder dysfunction and fatigue, adjusted for age (p<0.001, Table 2).

#### Bowel dysfunction and fatigue

There was a significant positive correlation between severity of fatigue (MFIS scores) and constipation (*p* < 0.001), adjusted for the confounding influence of EDSS. For a person with an EDSS of 1 and a MFIS score of 0, the baseline probability (risk) of constipation was 17% (0.169 (95% CI, 0.096 to 0.291)). The risk of constipation increased by 1.7% for every 1 point increase in MFIS (1.017 (95% CI, 1.008 to 1.026)) and for a 10 point increase in the fatigue score this would produce an 18% increased risk (1.017^10^). A significant positive relationship was also found between fatigue and the severity of faecal incontinence measured with the RFIS score (p<0.001), adjusted for level of disability (Fig 3C, Table 2). Similarly, there was a significant positive relationship between the severity of faecal incontinence (RFIS) and fatigue (p<0.001), adjusted for age (Table 2).

## Discussion

The aim of this study was to investigate the frequency of bowel and bladder symptoms in a sample of participants with MS, and to explore the relationship between the most frequently reported symptoms in in the Australian MS population–fatigue, disability, and bowel and bladder problems. Our study indicates that of 136 responders the frequency of bladder dysfunction in the MS population in New South Wales is 105/135 (77.8%), with frequency of bowel problems such as constipation and faecal incontinence 66/135 (48.9%) and 43/135 (31.9%), respectively. This agrees with a previous study by Wollin et al. [13] where 87% of 56 people with MS in Queensland, Australia, reported experiencing bladder problems, 59% constipation and 35% faecal incontinence. These rates are lower than reported in Victoria, Australia, with Khan et al. [11] reporting that 95.7% of their sample of 73 people with MS experienced some form of urinary problems. Prevalence of faecal incontinence (14%) was lower than reported here. However, it is important to note that both of these studies used different measurement scales to those employed here; thus, discrepancies in reported frequency rates of bladder and bowel problems across different parts of Australia should be interpreted with caution. Frequency rates of bladder and bowel dysfunction in our surveyed MS population are also similar to those reported in MS populations in other parts of the world.[7, 10, 12] Similarly, our finding that bladder problems, bowel problems and fatigue were significantly associated with level of disability (measured by EDSS scores) agrees with previous studies that have also documented a higher prevalence of bladder/ bowel problems and fatigue in those with higher levels of disability.[6, 11, 30, 31]

According to the Multiple Sclerosis International Federation, there are, on average, twice as many women as men with MS globally[1]; which is well- reflected in our study population (66.2% female). This report also stated that 85% of the global MS population were diagnosed with relapsing-remitting MS, of which 80% would progress to secondary progressive MS. Approximately 75% of our cohort had a primary diagnosis of either relapsing-remitting or secondary progressive MS, again closely reflecting the global MS population. Whilst the mean age of MS onset in the global population is around 30 years of age, this figure was slightly higher in our study population (38 years of age).

We also found a significant positive correlation between the severity of fatigue and bladder and bowel problems in our surveyed MS population. Although fatigue is one of the most common symptoms experienced by people with MS, with many plausible explanations,[1, 15] the possibility that significant bladder and bowel dysfunction may cause fatigue remains understudied. For instance, nocturia could result in disturbed sleep and reduced quality of sleep, contributing to daytime fatigue.[32] Furthermore, urinary symptoms such as urgency, frequency and incontinence may lead to reduced fluid intake to alleviate bladder problems, worsening hydration status which has been found to correlate with higher levels of fatigue.[33] The significant relationship between bowel dysfunction and fatigue may be explained by a reinforcing feedback loop previously illustrated by Crayton et al.,[34] in which fatigue results in a reduced ability to exercise, thereby exacerbating symptoms of constipation. Worries around bowel and bladder problems may also contribute to mental fatigue, thus worsening symptoms of fatigue experienced in people with MS.[13, 35] In turn, worsened fatigue may make effective toileting more difficult.[13, 34] Feelings of depression giving rise to fatigue as an associated symptom must also be considered when examining the relationship between bladder/ bowel difficulties and fatigue,[10, 34, 36] while medications that are used to treat urinary problems in people with MS can also have sedative effects.[37]

## Limitations

Our results are based on responses from 136 people with MS recruited from a single MS centre in Sydney. As these people were at a stage in their disease progression that their MS symptoms had to be actively managed, our sample may not be representative of the entire MS population in New South Wales, or Australia. Of note, 72.1% of our sample population had EDSS scores ≥5; this proportion of MS participants with moderate to severe disability is higher than that documented in similar studies.[30] A mean disease duration of 18 years and average age of 56 also suggests the sample may be bias towards a more severe level of disability. Given the correlation between higher disability levels and severity of bowel and bladder problems in persons with MS, it is possible that the frequency of bowel and bladder dysfunction in our study is overestimated. To minimise recruitment bias, a suitable next step would be to conduct this study at MS clinics located at different geographical locations in Australia. It is also possible that the group with highest levels of disability (EDSS scores 8–10) was underrepresented in our study, as people with a high disability status may be less likely to be able to attend the MS centre. Furthermore, response bias in the form of underreporting of bladder and bowel problems has been noted in previous studies and may be present here.[7, 13, 15] Given that people with MS often experience concurrent symptoms such as cognitive impairment, memory loss and impaired sensation, it is also possible that the accuracy of these self-reported data may be compromised in some cases.

## Conclusion

This is the first study to describe the frequency of bladder and bowel problems in the MS population in New South Wales, Australia. Our results indicate that the frequency of these symptoms in our select population are comparable to those reported in other states of Australia, and in other parts of the world. Notably, this study also presents new information about the correlation between bladder/ bowel function and fatigue in people with MS, which has not been previously highlighted in the literature. Further research in this area is needed to explore the cause-effect relationship between these MS symptoms, in order to augment current management strategies for people with MS. Our preliminary findings suggest that there is an increasing need for a multimodal approach to the treatment of MS, given the interconnectivity of various MS symptoms.

## Supporting information

S1 Table Raw_data_msstudy_plos. Raw data used to create all results presented in the manuscript.
(XLSX)

S2 Table msstudy_data_dictionary. Data dictionary for file “Raw_data_msstudy_plos”.
(XLSX)
